# Prognostic Impact of Nutritional Status on Overall Survival and Health-Related Quality of Life in Men with Advanced Prostate Cancer

**DOI:** 10.3390/nu15041044

**Published:** 2023-02-20

**Authors:** Luka Cavka, Maja Pohar Perme, Nada Rotovnik Kozjek, Bostjan Seruga

**Affiliations:** 1 Division of Medical Oncology, Institute of Oncology Ljubljana, Faculty of Medicine, University of Ljubljana, Zaloska Cesta 2, 1000 Ljubljana, Slovenia; 2 Department of Oncology, University Medical Center Maribor, Ljubljanska Ulica 5, 2000 Maribor, Slovenia; 3 Institute for Biostatistics and Medical Informatics, Faculty of Medicine, University of Ljubljana, Vrazov trg 2, 1000 Ljubljana, Slovenia; 4 Department of Clinical Nutrition, Institute of Oncology Ljubljana, Zaloska Ulica 2, 1000 Ljubljana, Slovenia

**Keywords:** nutritional risk, malnutrition, metastatic castrate-resistant prostate cancer, outcomes

## Abstract

Purpose: Prognostic role of nutritional status (NS) in patients with metastatic castrate-resistant prostate cancer (mCRPC) is unknown. We hypothesized that patients’ NS at the presentation of mCRPC is prognostic for health-related quality of life (HRQoL) and overall survival (OS). Methods: We conducted a prospective observational study in mCRPC patients. At enrollment, we allocated each patient into one of four NS categories: (i) well-nourished (WN), (ii) nutritional risk without sarcopenia/cachexia (NR), (iii) sarcopenia, or (iv) cachexia. We sought the prognostic role of the NS for OS and HRQoL by regression models. Results: 141 patients were included into our study. When compared to WN patients, those with NR and cachexia had a higher chance of worse HRQoL (OR 3.45; 95% CI [1.28 to 9.09], and OR 4.17; 95% CI [1.28 to 12.5], respectively), as well as shorter OS (HR 2.04; 95% CI [1.19 to 3.39] and HR 2.9; 95% CI [1.56 to 5.41], respectively). However, when accounting for possible confounding factors, we could not prove the significant importance of NS for chosen outcomes. Conclusions: Suboptimal NS might be an unfavorable prognostic factor for HRQoL and OS. Further interventional studies focusing on therapy or prevention are warranted.

## 1. Introduction

Prostate cancer is the second most common cancer in men, with an annual incidence of almost 1.4 million worldwide. About 20–30 percent of men with prostate cancer develop metastases and eventually die of this disease [1]. A late stage of prostate cancer is metastatic castrate-resistant prostate cancer (mCRPC), characterized by disease progression despite androgen deprivation therapy (ADT). Usually, disease burden gradually increases, and health-related quality of life (HRQoL) deteriorates in these patients over time [2]. In the last decade, we have seen a dramatic improvement in managing patients with advanced disease [3]. There is now strong evidence available from several phase III clinical trials showing that new anticancer therapies improve overall survival (OS) and HRQoL in patients with advanced prostate cancer [4].

Well known disease-related unfavourable prognostic factors in this disease are: high Gleason score, short time interval between radical treatment and recurrence, short prostate-specific antigen (PSA) doubling time, highly elevated PSA, extensive disease volume, presence of visceral metastases, presence of neuroendocrine histology and heavy burden of symptoms [5]. Our previous work demonstrated that suboptimal NS is associated with impaired HRQoL in patients with mCRPC at the presentation of their disease (see Table S1) [6]. Usually, patients with advanced prostate cancer have a long disease course of several years, during which they are exposed to various systemic therapies, including long-term androgen deprivation therapy (ADT), chemotherapy, and corticosteroids [7]. Both metastatic prostate cancer and its treatment can contribute to the gradual and insidious development of malnutrition and cachexia [8]. Malnutrition could affect survival and contribute to poorer tolerability of cancer treatment [9,10]. However, in contrast to unfavorable disease-related prognostic factors potential detrimental effect of malnutrition on patients’ lives may be potentially prevented or mitigated by nutritional interventions and physical activity.

Here, we hypothesize that NS at the presentation of mCRPC (i.e., at baseline) is prognostic for short-term HRQoL six months later and OS in patients with early mCRPC.

## 2. Patients and Methods

We designed a prospective observational study based on the guidelines provided by the international initiative EQUATOR (Enhancing the QUAlity and Transparency Of health Research) Network protocol (i.e., The Strengthening the Reporting of Observational Studies in Epidemiology STROBE statement) [11].

## 3. Study Design

As previously reported, all consecutively referred patients with early mCRPC to medical oncologists at the Institute of Oncology Ljubljana in two year period from July 2016 to July 2018 were evaluated for participation in this study [6]. We aimed to determine the prognostic value of baseline NS for HRQoL assessed six months after the inclusion into the study and for OS. Based on predefined exclusion criteria, we did not include patients with (i) cognitive impairment, (ii) Eastern Cooperative Oncology Group performance status ≥ 3, (iii) previous nutritional counseling within the last six months, (iv) inserted heart device (at the time of recruitment, it was the contraindication for bioimpedance analysis) and (v) unwillingness to participate. All participants provided informed consent. Written informed consent was obtained from all patients. The study was in accordance with the ethical standards laid down in the 1964 Declaration of Helsinki [12]. The approval was granted by the Medical Ethics Committee of Slovenia.

## 4. Assessment of the NS

**Figure 1:**
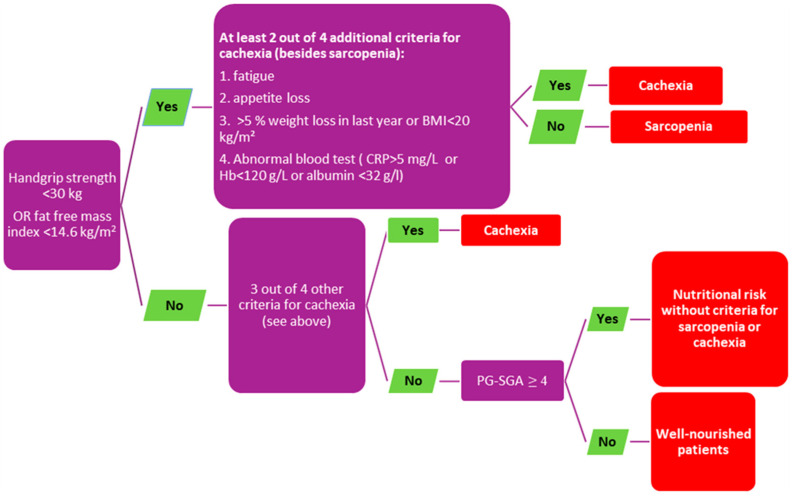
Algorithm for allocation in nutritional status category. (BMI body mass index, CRP C-reactive protein, Hb hemoglobin, PG-SGA patient generated subjective global assessment).

Based on clinical, laboratory, and patient self-reported criteria, we defined the well-nourished category (WN) and three suboptimal NS categories: nutritional risk without criteria for cachexia/sarcopenia (NR), sarcopenia, and cachexia. For the allocation process, we applied the algorithm that was previously described (Figure 1) [13].

## 5. HRQoL Assessment

We assessed the HRQoL by the validated questionnaire “Functional Assessment of Cancer Therapy” (FACT-P). It consists of generic questions about HRQoL in cancer patients and a 12-items prostate cancer-specific subscale. The score for each patient can range from 0 to 156 points, and the higher score reflects better HRQoL [14]. The HRQoL measurements Licensor FACIT Organization gave us the grant permission to use the FACT-P questionnaire.

## 6. Statistical Analysis

Some patients were not available for assessment of their HRQoL six months after enrollment for disease-related (i.e., early death, unsolvable disease progression) or unknown reasons. As unavailability of these patients for assessment might have an impact on results of our study (HRQoL), we considered their exclusion from the analysis inappropriate. Instead, we tried to address the problem of missing data. Firstly, we attributed to patients not available for assessment for disease-related reasons. HRQoL score of 0. In this case the distribution of numerical HRQoL scores was not normal and linear regression model was not appropriate for the analysis (Supplementary Figure S1). Therefore, the logistic regression model was used. The numerical score of the FACT-P questionnaire was transformed into a categorical variable, and two cut-off scores (50% and. 75% of the total possible FACT-P score) were considered for the dichotomization into two values (favorable vs. poor HRQoL). Based on the more real distribution of values (see Figure S2), the cut-off at 75% of the total score (i.e., 104 points) was used for the analysis and was further validated on our previous results 6 (Supplementary Tables S1 and S2). In the sensitivity analysis, 21 (14.9%) patients who were not available for assessment of HRQoL for unknown reasons were analyzed according to two scenarios: (i) they were allocated either into the favorable HRQoL group (i.e., best-case scenario) or (ii) into the poor HRQoL group (i.e., worst-case scenario). In fact, the outcome in both scenarios is the estimate of HRQoL, consisting of real (available data) and attributed values (unavailable data). Such approach allowed us to assess to what extent the missing data might have an impact on our results. The model of logistic regression was adjusted for a patient- and disease-related factors, which could be possible confounders: (i) duration of ADT, (ii) Charlson index of comorbidity, (iii) pain according to the visual analog scale (VAS), (iv) age, (v) the presence of visceral metastases, and (vi) laboratory measures (hemoglobin level and serum PSA level) [15,16].

The survival status of all enrolled patients was retrieved from the Cancer Registry of the Republic of Slovenia (cut-off date 20 June 2021). The OS was estimated by the Kaplan–Meier method. We employed the Cox regression model to assess the prognostic impact of NS on OS. Possible confounders mentioned above were included into the multivariate Cox model.

For the logistic and Cox regression models, odds ratios (ORs) and hazard ratios (HRs) and their 95% confidence intervals (95% CIs) are provided, respectively. *p*-values of <0.05 were deemed statistically significant. No adjustments for multiple comparisons were made.

## 7. Results

### Patients’ Characteristics

**Figure 2:**
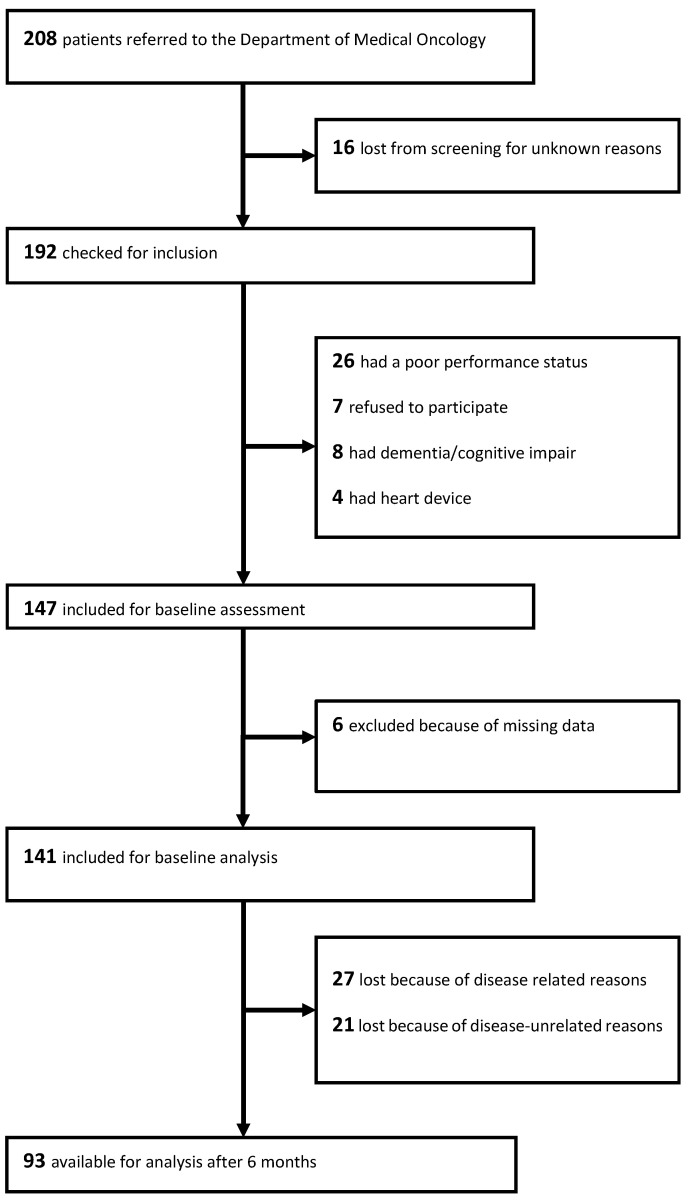
Flow chart of patients included into the study.

As previously described, we screened 208 patients and enrolled 141 patients in this study 6. Of these, 93 (66%) were evaluated for the HRQoL at the assessment six months later; there were missing data for disease-related reasons and unknown reasons in 27 (19.1%) and 21 (14.9%) patients, respectively (Figure 2). At the cut-off date 102 (72%) patients were dead.

The median age of patients was 74.1 years (IQR 68.6–79.4 years), and 18 (13%) had visceral metastases (for a detailed description of patients’ characteristics, see Supplementary Table S3). As previously reported, 59 (41.8%) patients were WN, followed by 24 (17%), 42 (29.8%), and 16 (11.3%) patients with NR, sarcopenia, and cachexia, respectively 6. Based on the detailed nutritional examination, we found 32 (22.7%) patients with appetite loss as well as 36 (25.5%), 42 (29.7%), 68 (48.2%), 2 (1.4%), 56 (25.7%), 2 (1.4%), 40 (28.4%), 2 (1.4%), and 63 (44.7%) with fatigue, low hemoglobin level, abnormal C-reactive protein (CRP), low albumins, low handgrip, low Fat-Free Mass Index (FFMI), more than 5% weight loss in last six months, Body Mass Index (BMI) < 20 kg/m^2^ and Patient-Generated Subjective Global Assessment score (G-SGA > 4), respectively (detailed patients’ characteristics at baseline are summarized in Table S3).

Overall, 137 (97.1%) patients received at least one line of potentially life-prolonging systemic anticancer therapy, which may also maintain or improve HRQoL. The distribution of the lines and type of therapies for each nutritional category is presented in Table 1. Treating oncologists prescribed enzalutamide substantially more frequently as compared to abiraterone acetate in patients with suboptimal NS but not in WN patients: NR (41.7% vs. 25%), sarcopenia (59.5% vs. 14.3%), cachexia (37.2% vs. 25%) and WN (37.3% vs. 30.1%), respectively.

## 8. Prognostic Role of Baseline NS for Estimated HRQoL

**Figure 3:**
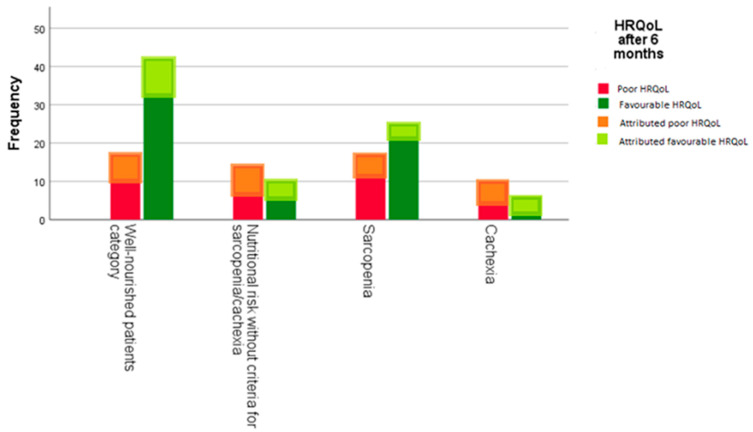
Distribution of actual and attributed HRQoL data six months after enrollment in each NS category according to the best-case scenario (all 21 patients not available for assessment for unknown reasons were attributed favorable HRQoL and all 27 patients not available for assessment for disease-related reasons were attributed unfavorable HRQoL).

Figure 3 presents actual and attributed HRQoL in each NS category, including attributed data for patients not assessed for HRQoL due to unknown reasons according to the best-case scenario. The WN patients have the lowest odds of having poor estimated HRQoL compared with other groups (Table 2). In the model of univariate logistic regression, we found that patients with NR and cachexia had a significantly higher chance of worse estimated HRQoL than WN patients (OR 3.45; 95% CI [1.28 to 9.09] and OR 4.17; 95% CI [1.28 to 12.5], respectively). Although there was a similar numerical trend for sarcopenia, this association was not statistically significant (Table 2). In the multivariate model trend for the negative association between NR, cachexia, and estimated HRQoL remained, but statistical significance was lost. Pain at baseline was the only significant negative predictor for estimated HRQoL at six months (Supplementary Table S4). When considering the worst-case scenario, results have not been substantially different (Supplementary Figure S3).

## 9. Prognostic Role of Baseline NS for OS

**Figure 4:**
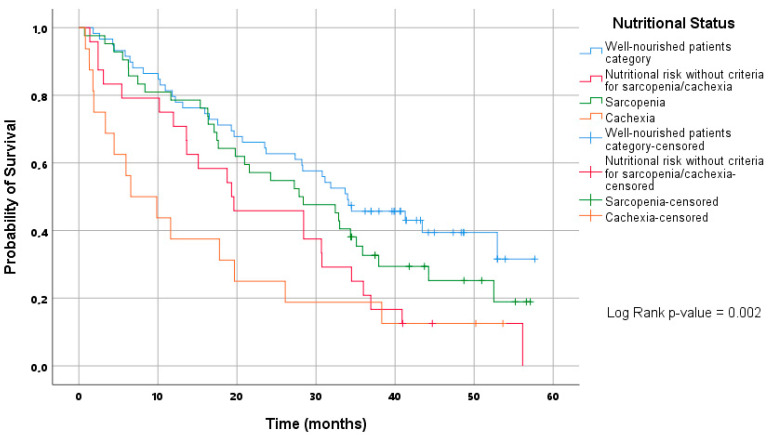
Kaplan Mayer curves of overall survival according to nutritional status.

The Kaplan-Meier curves show that WN patients have better OS than those within other nutritional categories (Figure 4). Suboptimal NS categories are associated with the worse OS when compared to the WN patients’ category, and for NR and cachexia, this association was statistically significant (HR 2.04; 95% CI [1.19 to 3.39] and HR 2.9 [1.56 to 5.41], respectively) (Table 3 and Table S5). However, we could not prove the significance of the NS category for OS when accounting for potential confounding factors.

## 10. Discussion

Malnutrition in cancer patients may be caused by various factors, including metabolic changes related to cancer, adverse effects of treatment, insufficient food intake, the inefficiency of healthcare systems, and psychosocial issues. In patients with advanced prostate cancer, malnutrition can develop gradually and insidiously over time. The impact of malnutrition and nutritional intervention on crucial aspects of lives of patients with mCRPC such as length and quality of life has not been elucidated yet. We previously reported that 58.2% of patients have suboptimal NS at the presentation of their mCRPC [6]. Results of our current analysis suggest that suboptimal NS at the presentation of mCRPC might be prognostically unfavorable for both short-term HRQoL and OS.

Recent advances in oncology and supportive care led to decreased morbidity and mortality of cancer patients. Therefore, HRQoL and its potential association NS with is becoming increasingly important. The HRQoL in cancer patients is a subjective multidimensional construct that represents the patients’ psychosocial well-being and functional status. It also reflects a subjective perception of disease symptoms and adverse effects burden [17].

As the oncology community is progressively paying more attention to improve quality of life of cancer patients, the instruments to assess the HRQoL should be utilized in clinical practice more frequently. Traditionally, the concept of HRQoL was focused on drugs’ adverse effects/disease symptoms management. Healthcare systems should provide enough time for a routine clinical examination to sufficiently address and cover those issues; optimal supportive care is a foundation of the quality cancer care. However, nowadays the concept HRQoL is much more widely understood than this. Not only that symptomatic non-life-threatening adverse events are under-detected and under-reported by health professionals, some other important cancer care-related issues (e.g., nutritional care, addressing of fears, disease acceptance, drug compliance, evaluation of physical activity, mood disorders, etc.) are often beyond the scope of routine clinical assessment [18]. A more integrative approach in outpatient care is faced with time and other restrictions. A very convenient approach to fill this gap could be utilization of patient-reported outcomes measures (PROMs).

Contrary to the daily clinical practice where PROMs are replacing standard clinical assessments at least to some extent, FACT-P questionnaire was used in our study to objectively measure HRQoL outcome and not to tailor clinical decisions. Contrary to the evidence of the superiority of PROMs as compared to clinical examination [17], there was an impression that patients enrolled into our study sometimes did not understand FACT-P related questions appropriately, felt tired of similar questions, and occasionally gave the lowest score to domains that did not interfere with their general perception of satisfaction. Unsurprisingly, the expectations about the future life were often lowered among our patients with metastatic disease. Our observations may suggest that PROMs should be used in conjunction with traditional clinical examinations, not instead of them.

In our study, we used a paper-based questionnaire FACT-P to measure HRQoL. Patients with mCRPC are usually older, therefore use of electronic version of the FACT-P questionnaire would very likely not be feasible. However, there is a general trend to adopt digital technology in all age groups, enabling the integration of electronic evaluation of symptoms, adverse effects, and other vital issues via mobile applications and websites. Even more important is to select relevant questionnaire items, preferably dynamic, to address expected disease symptoms, adverse effects of drugs, the unique need of age/social groups, comorbidities, and drug compliance. [17]. Importantly, such approach could make an additional step towards personalized oncology.

A systematic review reported a negative correlation between weight loss and HRQoL in patients with cancer cachexia [19]. It is well known that nutrition-related chemotherapy-related symptoms, such as nausea, vomiting, constipation, and fatigue, may negatively affect the HRQoL of cancer patients [20]. A growing body of evidence consistently shows that sarcopenia increases the risk of toxicity of chemotherapy and targeted agents [21]. Our study found that suboptimal NS of patients with mCRPC is associated with poor HRQoL six months later (Table 2). Results of our study also suggest that systemic anticancer therapy, started in 87.5–100% of our patients with different NS, may not mitigate the unfavorable impact of malnutrition on the short-term HRQoL (Table 1). However, the statistical significance of the association between NS and HRQoL was lost in the multivariate model (Supplementary Table S4). After adjustments for possible confounding factors, only baseline pain level remained the independent predictor of poor HRQoL at six months.

The most commonly prescribed therapy in this population of elderly patients was the 2nd generation antiandrogens, such as the combination of abiraterone acetate and methylprednisolone or enzalutamide, not chemotherapy (Table 1). Despite being unaware of assigned NS categories treating oncologists prescribed enzalutamide substantially more frequently than abiraterone acetate in patients with suboptimal NS but not in WN patients. The main reason for this decision might be the inclination to avoid loss of skeletal muscle mass, a well-known side effect of corticosteroids. However, this is in contrast with the results of recent studies, which show that both enzalutamide and abiraterone acetate led to a comparable loss of skeletal muscle mass [22]. It is currently not clear whether the impact of anticancer therapy on HRQoL differs between different NS categories. One might expect that patients with severe malnutrition require a longer time for recovery after cancer treatment as compared to patients with borderline or mild malnutrition [23]. Further subset analyses in our cohort were not feasible due to the small sample size.

Studies have unequivocally demonstrated that the prognosis for cancer patients with weight loss is worse than that for weight-stable patients [21,24]. Moreover, it is known that sarcopenia is associated with worse OS independently of weight loss [25]. In our study suboptimal baseline NS was unfavorably associated with OS. However, the number of lines of potentially life-prolonging therapies and type of agents differed between NS categories (Table 1). In the multivariate analysis, the significant association between suboptimal NS and OS was lost.

Information about weight loss does not identify all relevant pathophysiologic changes of clinical importance. The diagnosis of malnutrition in everyday clinical practice is often inappropriately based on weight loss only. According to the definition of cancer cachexia, we should focus on the ongoing loss of skeletal muscle mass (with or without fat mass loss) [26]. However, the identification of patients with muscle loss is problematic because it is not recognized with BMI measurement only. Also, 40–60% of cancer patients are overweight or obese, according to the World Health Organization criteria (WHO). Even in metastatic disease, sarcopenic obesity is frequently overlooked [24,27]. The strength of our study lies in diagnosing the NS according to terminology for nutritional disorders [28]. We categorized NS by the algorithm based on body composition, muscle strength, and laboratory parameters (Figure 1).

In contrast to malnourished patients with other types of cancer, we observed relatively few patients with low BMI, FFMI, and serum albumin in our cohort of patients [29]. It seems that these parameters seem to have low sensitivity in the assessment of nutritional risk in patients with mCRPC. Therefore, for a more objective assessment of nutritional status, standardized methods for assessing malnutrition should be used (e.g., Patient-Generated Subjective Global Assessment [PG-SGA] questionnaire) [30]. In our study, patients with NR were identified by using the PG-SGA questionnaire and had a similar risk for impaired HRQoL and shorter OS as those with more severe malnutrition. This finding indicates that a simple questionnaire such as PG-SGA could identify patients at risk for malnutrition early when nutritional or other intervention might be the most beneficial. Also, the regular clinical use of new methods for body composition measurements, as densitometry, CT- and MR- imaging techniques, can contribute to better clinical assessment of quantity and quality of muscle mass as nutritional marker in this group of patients [31].

As multiple factors are responsible for the development of cachexia, loss of skeletal muscle mass cannot be fully reversed by conventional nutritional support alone. Therefore, a multimodality treatment approach, including oral dietary supplements, exercise, and anti-inflammatory medications, is optimal for preventing and treating malnutrition [32]. The first results of the randomized MENAC trial (a multimodal intervention of exercise, nutrition, and anti-inflammatory medication plus standard care vs. standard care alone), which evaluates such a multidimensional approach in patients with advanced lung and inoperable pancreatic cancer treated with chemotherapy, are eagerly awaited [33]. Even in the case of bone metastases, which are present in most patients with mCRPC physical activity is safe and recommended [34]. It also prevents osteoporosis and fatigue and improves mental health [35]. There is no doubt that the optimal approach for managing malnutrition is multimodal, so it is crucial to collaborate in providing additional supportive care such as nutritional counseling, psychotherapy, physical/occupational therapy, pain control, and caregiver education and support.

Our study has several shortcomings. First, we cannot claim that the interpretation of results would have been the same if we had used an alternative HRQoL questionnaire. The FACT-P questionnaire was explicitly established for comparing HRQoL within interventional clinical trials and not observational studies. Second, there is no unequivocal definition of NS in the nutritional science society. It is possible to define NS categories differently, leading to different conclusions. For example, one may choose Fearon’s cachexia criteria, despite proven superiority in the prognostic effect of Evans’ criteria [36]. Third, as 137 out of 141 (97.1%) of our patients received at least one approved systemic anticancer treatment, it is unlikely that adjusting our results for systemic therapy in the multivariate analysis would lead to different results for HRQoL. However, patients with sarcopenia and cachexia less frequently received two or more lines of systemic therapy, which may be associated with worse OS. Treating severely malnourished and cachectic patients with anticancer therapy may be inappropriate as it may lead to severe toxicity and early death. Therefore, adjusting our results for the number of lines of systemic therapy seems inappropriate. Fourth, assessment of HRQoL later in the course of the disease might lead to different results; however, such an approach would further increase the missing data problem. In future similar studies, one may diminish the missing data problem by integrating a mobile app into the follow-up [37]. Additionally, some other baseline characteristics such as mental health status could have impact on both HRQoL and OS. However, mental health was not in the focus of our study. In fact, patients with severe cognitive decline were excluded from our study as they could not reliably fill in the study questionnaires. Furthermore, as FACT-P questionnaire includes questions about the mood and comfort level results of our analysis reflect the impact of mental health status on HRQoL to some extent. Finally, our sample was relatively small, raising the concern of enhanced statistical variability, which might lead to the loss of statistical significance of our findings in the multivariate model (Supplementary Table S4). Similar studies with larger sample sizes are warranted to reach more reliable conclusions. Moreover, the inclusion of the assessment of NS into randomized clinical trials evaluating new anticancer therapies might give a definitive answer about the prognostic role of malnutrition for HRQoL and OS in patients with advanced cancer.

## 11. Conclusions

The professionally assessed NS might provide prognostic information about HRQoL and OS in patients with mCRPC and therefore warrants further study. Our results suggest that interventions that improve NS may be very important for patients with advanced prostate cancer.

## Figures and Tables

**Table 1:** Systemic treatment according to the line and type of therapy in each nutritional subgroup.

	WN (N = 59)	NR (N = 24)	Sarcopenia (N = 42)	Cachexia (N = 16)
1st line	N = 59 (100%)	N = 24 (100%)	N = 40 (95.2%)	N = 14 (87.5%)
	Docetaxel (11) ARSI (38) Cabazitaxel (0) Radium-223 (3)	Docetaxel (6) ARSI (16) Cabazitaxel (0) Radium-223 (2)	Docetaxel (5) ARSI (31) Cabazitaxel (0) Radium (4)	Docetaxel (1) ARSI (10) Cabazitaxel (0) Radium-223 (3)
2nd line	N = 42 (71.7%)	N = 15 (62.5%)	N = 22 (52.3%)	N = 7 (43.8%)
	Docetaxel (16) ARSI (14) Cabazitaxel (3) Radium-223 (6) Other * (3)	Docetaxel (7) ARSI (4) Cabazitaxel (1) Radium-223 (3) Other * (0)	Docetaxel (7) ARSI (10) Cabazitaxel (2) Radium-223 (3) Other * (0)	Docetaxel (1) ARSI (4) Cabazitaxel (0) Radium-223(2) Other * (0)
3rd line	N = 27 (45.7%)	N = 11 (45.8%)	N = 16 (38.1%)	N = 4 (25%)
	Docetaxel (5) ARSI (11) Cabazitaxel (9) Radium-223 (2) Other * (0)	Docetaxel (1) ARSI (3) Cabazitaxel (5) Radium-223 (2) Other * (0)	Docetaxel (2) ARSI (5) Cabazitaxel (6) Radium-223 (2) Other * (1)	Docetaxel (1) ARSI (0) Cabazitaxel (2) Radium-223 (1) Other * (0)
˃3 lines	N = 25 (42.4%)	N = 11 (45.8%)	N = 11 (26.2%)	N = 1 (6.2%)
	Docetaxel (3) ARSI (11) Cabazitaxel (7) Radium-223 (0) Other * (4)	Docetaxel (2) ARSI (4) Cabazitaxel (1) Radium-223 (2) Other * (2)	Docetaxel (2) ARSI (6) Cabazitaxel (1) Radium-223 (2) Other * (0)	Docetaxel (0) ARSI (1) Cabazitaxel (0) Radium-223 (0) Other * (0)

Abbreviations: ARSI Androgen Receptor Signaling Inhibitor (i.e., abiraterone acetate/enzalutamide), NS nutritional status, HRQoL health-related quality of life, NR nutritional risk without criteria for sarcopenia/cachexia, WNPC well-nourished patients’ category. * mitoxantrone, carboplatin, olaparib, cisplatin/etopozide.

**Table 2:** Prognostic role of the baseline NS category for short-term estimated HRQoL.

Variable	OR [95% CI]	*p*-Value
NR vs. WN	3.45 [1.28 to 9.09]	0.01
Sarcopenia vs. WN	1.69 [0.73 to 3.84]	0.22
Cachexia vs. WN	4.17 [1.28 to 12.5]	0.02

Abbreviations: NS nutritional status, HRQoL health-related quality of life, NR nutritional risk without criteria for sarcopenia/cachexia, WN well-nourished, CI, Confidence Interval, OR Odds Ratio.

**Table 3:** Prognostic value of the baseline NS for OS.

Variable	HR [95% CI]	*p*-Value
NR vs. WN	2.04 [1.19–3.49]	˂0.01
Sarcopenia vs. WN	2.21 [0.84–2.21]	0.21
Cachexia vs. WN	5.54 [1.56–5.41]	˂0.01

Abbreviations: NR nutritional risk without criteria for sarcopenia/cachexia, WNPC well-nourished patients’ category.

## Data Availability

Data are available upon reasonable request.

## Supplementary Materials

The following supporting information can be downloaded at: https://www.mdpi.com/article/10.3390/nu15041044/s1, Figure S1: Distribution of the FACT-P scores after six months; Figure S2: Distribution of HRQoL categories according to different cut-off values of the FACT-P scores; Figure S3: Distribution of the HRQoL categories at six months across nutritional categories according to the worst-case scenario; Table S1: Association between baseline NS category and baseline HRQoL using the logistic regression model (validation of cutoff); Table S2: Estimate of health related quality of life after six months according to best case/worst case scenario; Table S3: Patients’ characteristics at baseline; Table S4: Prognostic value of baseline NS for HRQoL at six months (best-case scenario); Table S5: Overall survival in various NS categories.

## References

[1] Sung H., Ferlay J., Siegel R.L., Laversanne M., Soerjomataram I., Jemal A., Bray F. (2021). Global Cancer Statistics 2020: GLOBOCAN Estimates of Incidence and Mortality Worldwide for 36 Cancers in 185 Countries. CA Cancer J. Clin..

[2] Bergius S., Torvinen S., Muhonen T., Roine R.P., Sintonen H., Taari K. (2017). Health-related quality of life among prostate cancer patients: Real-life situation at the beginning of treatment. Scand. J. Urol..

[3] Lowrance W.T., Breau R.H., Chou R., Chapin B.F., Crispino T., Dreicer R., Jarrard D.F., Kibel A.S., Morgan T.M., Morgans A.K. (2021). Advanced Prostate Cancer: AUA/ASTRO/SUO Guideline PART I. J. Urol..

[4] Kretschmer A., Ploussard G., Heidegger I., Tsaur I., Borgmann H., Surcel C., Mathieu R., de Visschere P., Valerio M., van den Bergh R.C.N. (2021). Health-related Quality of Life in Patients with Advanced Prostate Cancer: A Systematic Review. Eur. Urol. Focus.

[5] Lowrance W.T., Breau R.H., Chou R., Chapin B.F., Crispino T., Dreicer R., Jarrard D.F., Kibel A.S., Morgan T.M., Morgans A.K. (2021). Advanced Prostate Cancer: AUA/ASTRO/SUO Guideline PART II. J. Urol..

[6] Cavka L., Pohar Perme M., Zakotnik B., Rotovnik Kozjek N., Seruga B. (2021). Nutritional Status and Health-Related Quality of Life in Men with Advanced Castrate-Resistant Prostate Cancer. Nutr. Cancer.

[7] Parker C., Castro E., Fizazi K., Heidenreich A., Ost P., Procopio G., Tombal B., Gillessen S., ESMO Guidelines Committee (2020). Prostate cancer: ESMO Clinical Practice Guidelines for diagnosis, treatment and follow-up. Ann. Oncol..

[8] Cushen S.J., Power D.G., Murphy K.P., McDermott R., Griffin B.T., Lim M., Daly L., MacEneaney P., O’ Sullivan K., Prado C.M. (2016). Impact of body composition parameters on clinical outcomes in patients with metastatic castrate-resistant prostate cancer treated with docetaxel. Clin. Nutr. ESPEN.

[9] Planas M., Álvarez-Hernández J., León-Sanz M., Celaya-Pérez S., Araujo K., Abelardo García de Lorenzo on behalf of the PREDyCES researchers (2016). Prevalence of hospital malnutrition in cancer patients: A sub-analysis of the PREDyCES^®^ study. Support Care Cancer.

[10] Gellrich N.C., Handschel J., Holtmann H., Kruskemper G. (2015). Oral Cancer Malnutrition Impacts Weight and Quality of Life. Nutrients.

[11] von Elm E., Altman D.G., Egger M., Pocock S.J., Gøtzsche P.C., Vandenbroucke J.P., Initiative S. (2007). The Strengthening the Reporting of Observational Studies in Epidemiology (STROBE) statement: Guidelines for reporting observational studies. Lancet.

[12] Cavka L., Perme M.P., Zakotnik B., Kozjek N.R., Seruga B. (2020). 676P Prognostic role of nutritional status (NS) for health-related quality of life (HRQoL) in men with advanced prostate cancer. Ann. Oncol..

[13] Cella D., Petrylak D.P., Fishman M., Teigland C., Young J., Mulani P. (2006). Role of quality of life in men with metastatic hormone-refractory prostate cancer: How does atrasentan influence quality of life?. Eur. Urol..

[14] Roydhouse J.K., Gutman R., Keating N.L., Mor V., Wilson I.B. (2018). Proxy and patient reports of health-related quality of life in a national cancer survey. Health Qual. Life Outcomes.

[15] Morgans A.K., van Bommel A.C., Stowell C., Abrahm J.L., Basch E., Bekelman J.E., Berry D.L., Bossi A., Davis I.D., de Reijke T.M. (2015). Development of a Standardized Set of Patient-centered Outcomes for Advanced Prostate Cancer: An International Effort for a Unified Approach. Eur. Urol..

[16] Arndt V., Merx H., Stegmaier C., Ziegler H., Brenner H. (2004). Quality of life in patients with colorectal cancer 1 year after diagnosis compared with the general population: A population-based study. J. Clin. Oncol..

[17] Di Maio M., Basch E., Denis F., Fallowfield L.J., Ganz P.A., Howell D., Kowalski C., Perrone F., Stover A.M., Sundaresan P. (2022). The role of patient-reported outcome measures in the continuum of cancer clinical care: ESMO Clinical Practice Guideline. Ann. Oncol..

[18] Di Maio M., Basch E., Bryce J., Perrone F. (2016). Patient-reported outcomes in the evaluation of toxicity of anticancer treatments. Nat. Rev. Clin. Oncol..

[19] Wheelwright S., Darlington A.S., Hopkinson J.B., Fitzsimmons D., White A., Johnson C.D. (2013). A systematic review of health-related quality of life instruments in patients with cancer cachexia. Support Care Cancer.

[20] Lis C.G., Gupta D., Lammersfeld C.A., Markman M., Vashi P.G. (2012). Role of nutritional status in predicting quality of life outcomes in cancer—A systematic review of the epidemiological literature. Nutr. J..

[21] Ryan A.M., Power D.G., Daly L., Cushen S.J., Bhuachalla E.N., Prado C.M. (2016). Cancer-associated malnutrition, cachexia and sarcopenia: The skeleton in the hospital closet 40 years later. Proc. Nutr. Soc..

[22] Fischer S., Clements S., McWilliam A., Green A., Descamps T., Oing C., Gillessen S. (2020). Influence of abiraterone and enzalutamide on body composition in patients with metastatic castration resistant prostate cancer. Cancer Treat Res. Commun..

[23] Fearon K., Strasser F., Anker S.D., Bosaeus I., Bruera E., Fainsinger R.L., Jatoi A., Loprinzi C., MacDonald N., Mantovani G. (2011). Definition and classification of cancer cachexia: An international consensus. Lancet Oncol..

[24] Martin L., Senesse P., Gioulbasanis I., Antoun S., Bozzetti F., Deans C., Strasser F., Thoresen L., Jagoe R.T., Chasen M. (2015). Diagnostic criteria for the classification of cancer-associated weight loss. J. Clin. Oncol..

[25] Martin L., Birdsell L., Macdonald N., Reiman T., Clandinin M.T., McCargar L.J., Murphy R., Ghosh S., Sawyer M.B., Baracos V.E. (2013). Cancer cachexia in the age of obesity: Skeletal muscle depletion is a powerful prognostic factor, independent of body mass index. J. Clin. Oncol..

[26] Bruggeman A.R., Kamal A.H., LeBlanc T.W., Ma J.D., Baracos V.E., Roeland E.J. (2016). Cancer Cachexia: Beyond Weight Loss. J. Oncol. Pract..

[27] Ramos Chaves M., Boléo-Tomé C., Monteiro-Grillo I., Camilo M., Ravasco P. (2010). The diversity of nutritional status in cancer: New insights. Oncologist.

[28] Cederholm T., Barazzoni R., Austin P., Ballmer P., Biolo G., Bischoff S.C., Compher C., Correia I., Higashiguchi T., Holst M. (2017). ESPEN guidelines on definitions and terminology of clinical nutrition. Clin. Nutr..

[29] Cushen S., Power D., McEneaney P., McLaughlin P., Fitzpatrick F., Ryan A. (2013). A prospective investigation of nutritional status of ambulatory Irish oncology patients undergoing chemotherapy: Prevalence of malnutrition, cachexia, sarcopenia and impact on quality of life. Eur. J. Cancer.

[30] Guo Z.Q., Yu J.M., Li W., Fu Z.M., Lin Y., Shi Y.Y., Hu W., Ba Y., Li S.Y., Li Z.N. (2020). Survey and analysis of the nutritional status in hospitalized patients with malignant gastric tumors and its influence on the quality of life. Support Care Cancer.

[31] Baracos V.E., Arribas L. (2018). Sarcopenic obesity: Hidden muscle wasting and its impact for survival and complications of cancer therapy. Ann. Oncol..

[32] Fearon K.C. (2008). Cancer cachexia: Developing multimodal therapy for a multidimensional problem. Eur. J. Cancer.

[33] Solheim T.S., Laird B.J.A., Balstad T.R., Bye A., Stene G., Baracos V., Strasser F., Griffiths G., Maddocks M., Fallon M. (2018). Cancer cachexia: Rationale for the MENAC (Multimodal-Exercise, Nutrition and Anti-inflammatory medication for Cachexia) trial. BMJ Support. Palliat. Care.

[34] Campbell K.L., Cormie P., Weller S., Alibhai S.M.H., Bolam K.A., Campbell A., Cheville A.L., Dalzell M.A., Hart N.H., Higano C.S. (2022). Exercise Recommendation for People With Bone Metastases: Expert Consensus for Health Care Providers and Exercise Professionals. JCO Oncol. Pract..

[35] Galvao D.A., Newton R.U., Gardiner R.A., Girgis A., Lepore S.J., Stiller A., Mihalopolous C., Occhipinti S., Chambers S.K. (2015). Compliance to exercise-oncology guidelines in prostate cancer survivors and associations with psychological distress, unmet supportive care needs, and quality of life. Psycho-Oncol..

[36] Vanhoutte G., van de Wiel M., Wouters K., Sels M., Bartolomeeussen L., De Keersmaecker S., Verschueren C., De Vroey V., De Wilde A., Smits E. (2016). Cachexia in cancer: What is in the definition?. BMJ Open Gastroenterol..

[37] Grašič Kuhar C., Gortnar Cepeda T., Kovač T., Kukar M., Ružić Gorenjec N. (2020). Mobile App for Symptom Management and Associated Quality of Life During Systemic Treatment in Early Stage Breast Cancer: Nonrandomized Controlled Prospective Cohort Study. JMIR Mhealth Uhealth.

