# Supplementary material for: Prognostic Impact of Nutritional Status on Overall Survival and Health-Related Quality of Life in Men with Advanced Prostate Cancer
Source: Nutrients. 2023 Feb 20;15(4):1044. doi: 10.3390/nu15041044 (PMC9964768; doi:10.3390/nu15041044)
Supplement: Supplementary file 1 [file nutrients-15-01044-s001.zip › nutrients-2216336-supplementary.pdf]

Table S1. Association between baseline NS category and baseline HRQoL using the logistic regression model (validation of cutoff)

Variable	OR [95 % CI]	p-value
NR vs. WN	4.76 [1.79 – 4.76]	< 0.01
Sarcopenia vs. WN	4.76 [2.33 -11.1]	< 0.01
Cachexia vs. WN	43.47 [5.34 – 333.3]	< 0.01

Abbreviations: WN well-nourished patients, NR nutritional risk without criteria for sarcopenia/cachexia

Table S2. Estimate of health related quality of life after six months according to best case/worst case scenario

HRQoL after six months	Favorable HRQoL	Poor HRQoL
Best case scenario	82	59
Worst case scenario	60	81

Abbreviations: HRQoL health-related quality of life

Table S3. Patients` characteristics at baseline (n=141)

Variable	Median	IQR
PG-SGA (points)	3	2-7
BMI (kg/m^2)	26.9	24.5-30
FFMI (kg/m^2)	18.5	17.28-20.03
Handgrip strength (kg)	31	23-39
ADT (months)	24.4	13.7-50
Charlson index (score)	7	6-8
Pain (VAS score)	2	0-4
Age (years)	74.1	68.6-79.4
Hb (g/l)	131	117-142
PSA (ng/ml)	103.6	38.8-194.3

Abbreviations: BMI, Body Mass Index; *FFMI* Fat-Free Mass Index, *ADT* duration of androgen deprivation therapy, *VAS* visual analog scale for pain assessment, *Hb,* Hemoglobin; *PSA,* Prostate-Specific Antigen; *PG-SGA,* Patient Generated-Subjective General Assessment

Table S4. Prognostic value of baseline NS for HRQoL at six months (best-case scenario)

Variable	OR [95 % CI]	p-value
NR vs. WN	2.86 [0.92 to 9.09]	0.07
Sarcopenia vs. WN	0.85 [0.30 to 2.38]	0.77
Cachexia vs. WN	1.75 [0.37 to 8.33]	0.48
ADT (months)	1.04 [0.99 to 1.11]	0.11
Comorbidities (score)	1.03 [0.81-1.28]	0.84
Pain (VAS score)	1.28 [1.15 to 1.64]	<0.001
Age (years)	1.04 [0.98 to 1.11]	0.96
Visceral metastases (yes/no)	1.59 [0.54 to 4.76]	0.40
Hemoglobin (g/l)	0.99 [0.97 to 1.02]	0.76
PSA (ng/ml)	1 [0.998 to 1.02]	0.64

Abbreviations: NR nutritional risk without criteria for sarcopenia/cachexia, WN well-nourished patients, ADT duration of androgen deprivation therapy, VAS visual analog scale for pain, PSA prostate-specific antigen

Table S5. *Overall survival in various NS categories*

NS category	Median OS (months)	95 % CI for OS (months)
WN	34	23.63 to 44.37
NR	19.32	6.53 to 32.11
Sarcopenia	27.56	15.91 to 39.80
Cachexia	6.57	0 to 14.2

Abbreviations: NS nutritional status, NR nutritional risk without criteria for sarcopenia/cachexia, WN well-nourished patients

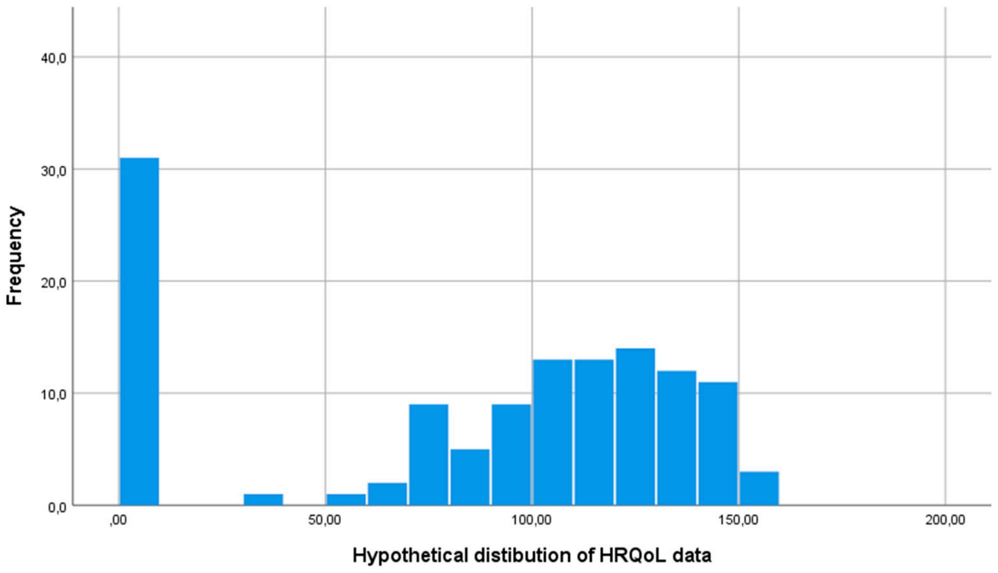

Figure S1. Distribution of the FACT-P scores after six months. Missed patients for disease-related reasons have attributed a score of 0, and patients missed for unknown reasons were excluded

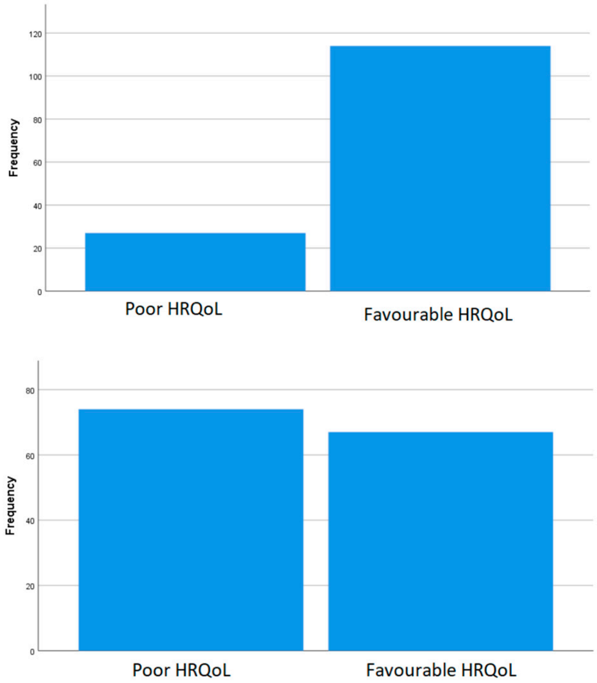

Figure S2. Distribution of HRQoL categories according to different cut-off values of the FACT-P scores; Upper chart: Testing for the cut-off at 50% (78 points); Lower chart: Testing for the cut-off at 75% (104 points)

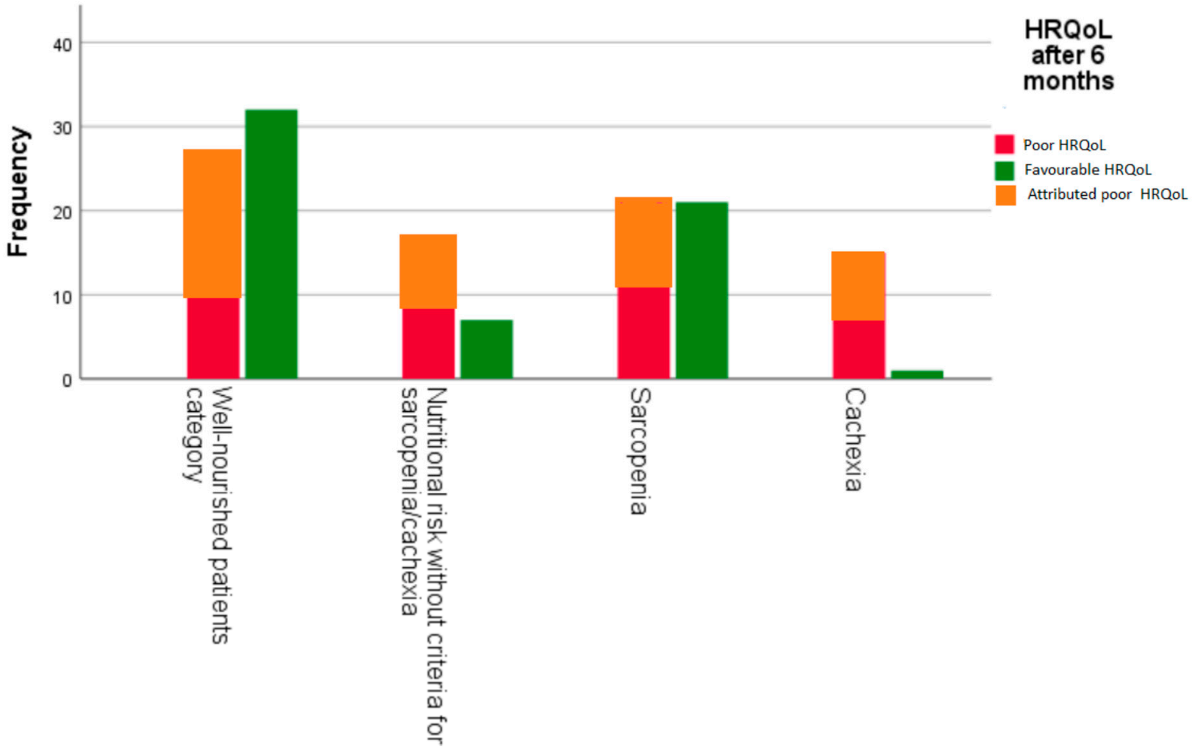

Figure S3. Distribution of the HRQoL categories at six months across nutritional categories according to the worst-case scenario
